# Thermodynamics of voltage-gated ion channels

**DOI:** 10.1007/s41048-018-0074-y

**Published:** 2018-11-16

**Authors:** Xuejun C. Zhang, Hanting Yang, Zhenfeng Liu, Fei Sun

**Affiliations:** 1 National Laboratory of Biomacromolecules, CAS Center for Excellence in Biomacromolecules, Institute of Biophysics, Chinese Academy of Sciences, Beijing, 100101 China; 2 College of Life Science, University of Chinese Academy of Sciences, Beijing, 100049 China

**Keywords:** Ion channel, Gating mechanism, Membrane potential, Voltage sensor, Sliding-rocking model, Inactivation

## Abstract

Ion channels are essential for cellular signaling. Voltage-gated ion channels (VGICs) are the largest and most extensively studied superfamily of ion channels. They possess modular structural features such as voltage-sensing domains that encircle and form mechanical connections with the pore-forming domains. Such features are intimately related to their function in sensing and responding to changes in the membrane potential. In the present work, we discuss the thermodynamic mechanisms of the VGIC superfamily, including the two-state gating mechanism, sliding-rocking mechanism of the voltage sensor, subunit cooperation, lipid-infiltration mechanism of inactivation, and the relationship with their structural features.

**Electronic supplementary material:**

The online version of this article (10.1007/s41048-018-0074-y) contains supplementary material, which is available to authorized users.

## Introduction

Information transmission in living cells is encoded through ion fluxes, resulting in complex spatiotemporal concentration patterns of ions. Being both a driving force and a result of these ion fluxes, the membrane potential is deeply intertwined with the dynamic distribution of ions across the membrane. Together, membrane potential and ion fluxes form an information network for regulation of cellular functions (Roux 1997). In this network, the transmembrane electrochemical potential is the major power source, and a variety of ions can be considered as the information carriers. Approximately 1/3–2/3 of cellular energy originating from ATP hydrolysis is consumed to maintain the membrane potential (Howarth *et al*. 2012), in addition to other cellular resources used to establish and preserve the network infrastructure. In fact, together with redox potential and ATP hydrolysis, membrane potential is one of the three major, interchangeable energy forms in all of living cells (Smith and Morowitz 2016). Without a properly maintained membrane potential, the cell would lose its essential information network. Similarly, ion channels, together with ion-sensing proteins as well as downstream effectors, are equally important, as they constitute basic functional nodes of such information network, perceiving, processing, and transducing signals encoded in ion fluxes (Jan and Jan 1989).

Ion channels are essential for transmembrane signaling and ionic homeostasis as well as regulating membrane polarization (Yellen 2002). They function in the plasma membrane, membranes of certain intracellular organelles, and other dynamic systems mediating cooperativities between organelles (Hosaka *et al*. 2017; Kintzer and Stroud 2017; Wei *et al*. 2016; Xu and Ren 2015; Yang *et al*. 2016). Ion channels are fundamentally different from another type of membrane proteins, namely transporters. While transporters are mainly responsible for transmembrane transport of metabolites and other substances such as nutrition uptake, ion channels are used for transducing ion-encoded information. In fact, the ions flowing through channels need to be replenished later by transporters or ion pumps. Such pumps are powered either directly by ATP hydrolysis or by transmembrane electrochemical potential of other substances (*e.g.*, protons). Moreover, an ion channel differs from a transporter in its ability to transduce its substrate ions at a rate a few magnitudes larger than that of a transporter. Importantly, the substrate translocation pathway of a canonical ion channel opens to both sides of the membrane simultaneously. In contrast, a typical transporter opens to only one side at a given time through the alternating access mechanism (Jardetzky 1966). As the ion flux through a channel is driven by an electrochemical potential, whose maintenance renders fully functioning transporters an essential prerequisite, the signaling function of channels presumably appeared in the evolutionary course later than active transporters; nevertheless, this order remains to be a subject of debate (Pohorille 2005; Saier 2016). Interestingly, certain types of transporters can be converted into channels with limited structural modifications, as exemplified by the relationship between proton-pump bacteriorhodopsins and the light-evoked channel-rhodopsin from motile microalgae (Deisseroth and Hegemann 2017) and by that between canonical ABC exporters and the ATP-gated channel CFTR (Liu *et al*. 2017). Since the kinetic rates (*e.g*., activation rate) of a channel are high compared to that of transporters, conformational changes in the functional cycle of a channel are likely to be relatively small. In addition, persistent opening of a channel would result in depletion of cellular resources and thus be detrimental to cell viability. Therefore, channels are tightly regulated in terms of both substrate selectivity and permeability, and thus remain in their open state only transiently. A plethora of mechanisms for regulating channels has been discovered, including the gating mechanisms by voltage (Yellen 2002), ligand binding (Miller and Aricescu 2014; Wei *et al*. 2016), pH (Thompson *et al*. 2008), phosphorylation (Liu *et al.* 2017), mechanical force (Haswell *et al*. 2011; Zhang *et al*. 2016), photon (Deisseroth and Hegemann 2017), and temperature (Memon *et al*. 2017). A given ion channel can be regulated through multiple gating mechanisms, thus participating in complex signaling networks.

As fundamental functional units on the membrane of excitable cells, voltage-gated ion channels (VGICs) are crucial for electrical signaling in nerves and muscle cells among others. The VGIC superfamily includes members of the K^+^-(K_V_), Na^+^-(Na_V_), Ca^2+^-, Cl^−^-, and proton-channel families (Jan and Jan 1989; Purves *et al*. 2001; Tu *et al*. 2018), and has been extensively characterized through functional and structural approaches. Because of the vast volume of research literature on VGICs, we confine ourselves here to discuss the relationship between their common structural features and thermodynamic bases of their common mechanisms such as gating and voltage sensing. For more extensive discussions on the structure and function of ion channels as well as their physiological and clinical implications, readers are referred to excellent reviews published earlier, for examples Refs. (Bagneris *et al*. 2015; Catterall 2000; Jan and Jan 1989; Kintzer and Stroud 2017; Tombola *et al*. 2005a; Yellen 2002; Yu *et al*. 2005).

## Overall structures of canonical VGICs

**Fig. 1:**
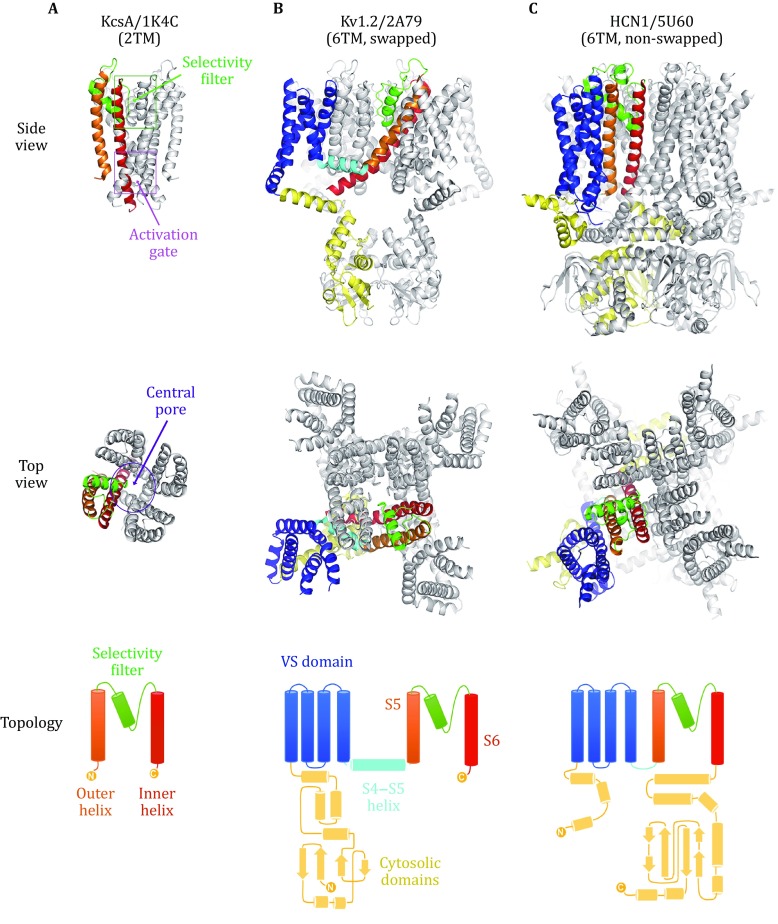
Structures of tetrameric ion channels. **A** 2TM channel of KcsA/1K4C (Zhou *et al*. 2001). **B** Swapped 6TM channel of Kv1.2/2A79 (Long *et al*. 2005). **C** Non-swapped 6TM hyperpolarization-activated channel of HCN1/5U6O (Lee and MacKinnon 2017). From the top to bottom are the side view, top view, and topology diagrams. Structural elements from one subunit are colored as the following: VS domain, *blue*; S4–S5 helix, *cyan*; S5 (outer) helix, *orange*; selectivity-filter, *green*; S6 (inner) helix, *red*; and cytosolic domains, *yellow*. The other three subunits of the tetramer are shown in *gray*

For the sake of the following mechanistic discussion, we briefly summarize the common structural features of canonical, tetrameric VGICs (or simply referred here to as ion channels), using K^+^ and Na^+^ channels as examples. A typical tetrameric ion channel is composed of four, identical (or homologous), symmetrically assembled, transmembrane subunits (or repeats) (Fig. 1). Each subunit of the homo-tetrameric channel contains either two or six transmembrane (TM) helices. In a 2TM subunit, such as the KcsA channel from *Streptomyces lividans*, the two TM helices are termed outer and inner helices (Doyle *et al*. 1998), and in a 6TM subunit, such as the rat K_V_1.2 channel, the TM helices are termed S1–S6 (Long *et al*. 2005). The S5 and S6 helices of the 6TM subunit are equivalent to the outer and inner helices of the 2TM subunit, respectively. In particular, the inner (S6) helices form a fourfold symmetrical, ion-conducting, central pore, and thus are called pore-lining helices. Therefore, the 6TM subunit contains, at its C-terminal region, a pore domain homologous to the 2TM subunit, with additional N-terminal S1–S4 helices forming a voltage-sensor (VS) domain. A subunit of the tetrameric 6TM channel either has its pore domain and VS domain in direct contact with each other or swaps its VS domain with neighboring subunits. In addition, two or four copies of the 6TM subunits may fuse into one peptide and become diversified between these repeats. The resulting pseudo-tetrameric channels contain only one or two such peptides (Kintzer and Stroud 2017; Yellen 2002). Certain channels employing more sophisticated regulatory mechanisms often contain additional domains, which may be significantly larger than the 6TM subunit itself (des Georges *et al*. 2016; Wei *et al*. 2016).

As it forms the central pore of the tetrameric ion channel, the 2TM subunit provides a suitable framework for understanding the common mechanisms of ion selectivity and gating in canonical tetrameric ion channels. From an evolutionary point of view, this subunit very likely represents an ancient, primordial form of currently existing ion channels (Yu *et al*. 2005). In support of this argument, the 2TM subunit constitutes a number of essential structural features of the central pore. For example, both the N- and C-termini of this domain are located on the cytosolic side, defining the overall topology of the transmembrane helices. As one of the most noticeable structural features of all tetrameric ion channels, four inner (S6) helices form an “inverted teepee”-like core structure of the channel (viewed in the conventional orientation in which the cytosolic side is located at the bottom). This central core consists of three major layers: (1) The C-terminal segment of S6 helices bundle together, forming a right-handedly crossing, size-adjustable pore termed activation-gate, in the cytosolic 1/3 region of the central pore. The inside wall of the activation-gate is typically lined with three rings of hydrophobic residues (Doyle *et al*. 1998), and this hydrophobicity is critical for the gating process (discussed below). (2) A short pore-helix (also called P-helix) and a pore-loop exist between S5 and S6 in the primary sequence of each subunit. At the level of the outer leaflet of the lipid bilayer, these P-helices and pore-loops fold inside the splayed part of the teepee-like structure. The pore-loop is stretched between the re-entrant P-helix and the N-terminus of S6. Four pore-loops form a fourfold (pseudo-)symmetrical, polar selectivity-filter, in the extracellular 1/3 of the central pore. In K^+^ channels, only the backbone carbonyl groups of the pore-loop contribute to the binding of the substrates (McCoy and Nimigean 2012). For Na^+^ and Ca^2+^ channels, an additional short helix, P2, is further inserted between the pore-loop and S6 in the primary sequence, and the selectivity-filter is formed by a mixture of side-chain polar groups and backbone carbonyl groups from the pore-loop. (3) An overall hydrophobic central cavity, which has a diameter larger than 10 Å, is located between the activation-gate and selectivity-filter in the tetrameric structure, constituting the middle 1/3 region of the channel pore. This central cavity readily accommodates a cation together with its first hydration shell (roughly 8 Å in diameter). In K^+^ channels, this cavity is shown to be a strong ion binding site at the entrance of the selectivity-filter (Zhou *et al*. 2001). In addition, a negatively charged vestibule is often found around the entrance of a cation channel, functioning as a buffer zone to increase the local concentration of substrate cations (Payandeh *et al*. 2011). The structures of both the selectivity-filter and central cavity do not change significantly during channel gating (Bagneris *et al*. 2015; McCusker *et al*. 2012). Therefore, a consensus has been formed that most gating mechanisms are likely to operate through adjusting the physical and chemical properties of the hydrophobic activation-gate, which will be discussed in details below.

## Ion movement and conductance

Movement of the permeant substrate ions through a channel is driven by (and thus consumes) the transmembrane electrochemical potential of the substrates, including components of the membrane potential ∆*Ψ*_M_ (≡*Ψ*_in_ −*Ψ*_out_) and/or concentration gradient of the substrate ions. The concentration gradient is commonly represented as the Nernst potential, *V*_N_ (≡(*RT*/z*F*)·ln([*S*]_in_/[*S*]_out_), where *z* is the charge valent of the substrate ion, and *RT*/*F* ≈ 25 mV at 20 °C). Therefore, the overall effective transmembrane voltage, ∆*Ψ*′ (≡∆*Ψ*_M_ + *V*_N_), represents the sole energy source driving substrate movement in nearly all ion channels.

Ion channels are arguably the best studied class of membrane proteins, largely because of the well-established biophysical techniques available for functional investigation. Among these techniques, patch-clamp electrophysiology represents the “gold standard” for monitoring channel current, and provides quantitative information on the relationship between the transmembrane potential and ion movement (*i.e.*, the electric current). In a patch-clamp study, the external voltage source, which replaces the membrane potential ∆*Ψ*_M_, is controlled experimentally. After compensating *V*_N_, the ratio of the electric current (*I*) to externally applied voltage (*V*) at each point of the recorded *I*–*V* curve is, by definition, the macroscopic conductance of the channels (*i.e.*, *G*(*V*) as a function of voltage) (Fig. 1A). (Conductance is not to be confused with the Gibbs energy introduced below, also denoted as *G*). Once opened, each single channel is assumed to display a unitary single-channel conductance (*g*_sc_), and its value was estimated to be in the range of ~2–30 pS for K^+^ channels (Gutman *et al*. 2005). At a 100-mV ∆*Ψ*_M_, this range in conductance translates into a transient transporting rate of ~10^7^ ions per second. In some rare cases, the *g*_sc_ appears to have multistep values, corresponding to distinct substrate selectivity and presumably being resulted from multi-conformations of the selectivity-filter (Zheng and Sigworth 1998). The macroscopic conductance is, thus, the product of the number of opened channels and *g*_sc_. The assumption of unitary *g*_sc_ for a given ion channel implies a microscopic two-state model, in which a single channel is either fully closed or fully opened rather than partially opened. Such a two-state model of channel gating has been supported by results from numerous voltage-clamp studies on a variety of single tetrameric ion channels (*e.g.*, in Ren *et al*. 2001). Therefore, the normalized macroscopic conductance (*G*/*G*_max_, where *G*_max_ represents the maximum conductance under a given experimental setup) equals to the channel-open probability (Zarrabi *et al*. 2010).

Hypothetically, an ideal linear *I*–*V* relationship in a whole-cell electrophysiological experiment would indicate that the channels conduct substrate ions with a voltage-independent conductance. However, instead of displaying a perfect linear *I*–*V* relationship, the macroscopic conductance of real channels always exhibits certain ∆*Ψ*_M_-dependency for a number of reasons. First, no such linear relationship could be maintained if the magnitude of the applied voltage approached infinity. In fact, the ion current running through a single channel is limited by the diffusion rate of substrate ions from the surrounding into the buffer zone. Moreover, the structure as well as the function of a protein is strongly influenced by its environment. The most important environmental factor for an integral membrane protein, such as a channel, constitutes the membrane lipid bilayer. By adjusting the equilibrium position relative to the ∆*Ψ*_M_-bearing membrane of a charge-carrying channel, the kinetic barrier of the channel and thus its *g*_sc_ value may gradually change with changing of the membrane potential. In extreme cases, this type of regulation results in rectification of the *I*–*V* curve. More generally, as long as it is not bound with the ∆*Ψ*_M_, any charge-carrying membrane protein has the potential to function as a ∆*Ψ*_M_-sensor. Nevertheless, the readout may not be always as evident as an ion channel in a voltage-clamp assay. While the theoretical possibility of such effects of ∆*Ψ*_M_ on membrane proteins had been discussed several decades ago (Hill 1985), this type of interaction between membrane proteins and the ∆*Ψ*_M_ is still not widely appreciated, especially in the field of studying the structures of membrane proteins. We argue that many long-range effects of mutations involving change of charges in membrane proteins might be explained as a consequence of their interaction with ∆*Ψ*_M_. For example, a single-point mutation, K1237E, in the filter region of repeat-IV of rNa_V_1.4 resulted in a change of double charges, and this variant showed ultra-slow inactivation (Todt *et al*. 1999). This K1237E mutation probably has two effects, namely (1) “horizontal” repulsion between existing and introduced acidic residues inside the selectivity-filter, and (2) in the presence of ∆*Ψ*_M_, a “vertical” electrostatic force (on the extra negative charges) which is likely to have a long-range effect on the conformation of the activation-gate. In addition to the “gradual” change of *g*_sc_ with ∆*Ψ*_M_, the activation-gate of a VGIC is able to abruptly switch between close and open states in response to the state transition of the VS domains. Taken together, the apparent conductance of a single ion channel is its “weakly” ∆*Ψ*_M_-dependent single-channel conductance, *g*_sc_, modulated by the step-function of the VS activation.

Whereas ∆*Ψ*_M_ directly exerts its power through interaction with the electric charges of the substrate ions, the other component of ∆*Ψ*′, *V*_N_, originates from the chemical potential (*i.e*., concentration gradient) of the substrate ions and appears to act indirectly. Mathematically, a force is defined as the decrease in energy across a specific distance. It is in this sense that the thermodynamic force of *V*_N_ becomes comparable with the deterministic force of ∆*Ψ*_M_. Let us first look into the deterministic force of ∆*Ψ*_M_. In the steady open state of the channel, the electrostatic energy of the ions is utilized to overcome the friction inside the selectivity-filter, thus dissipating as heat. Since the single-channel conductance remains constant, the ion movement (*I*) is linearly proportional to the electrostatic force applied to the ions (*V*), following the macroscopic Ohm law. Thus, the friction/damping coefficient (*i.e.*, the ratio of the applied force to the speed of ion movement (Qian and Kou 2014)) remains constant at the microscopic level. Secondly, we look into the statistical force of *V*_N_. An ion that binds to the entrance of the selectivity-filter does not directly sense the concentration of the same type of ions at the other end of the filter and beyond. Rather, this ion senses the gradient through electrostatic repelling between ions. This electrostatic force is propagated along the selectivity-filter in a manner similar to Newton’s cradle. Interestingly, substrates of almost all known selective channels are electrically charged. For simplicity, we consider only the electric potential between the two ends of the selectivity-filter of an ideal channel. In such an ideal channel, the two terminal binding sites are specific to the substrate ions, with negligible affinity to other ions; otherwise complicated competition with (and/or inhibition by) other substances must be included for kinetic consideration. In addition, the substrate specificity is determined not only by affinity at the two ends but also by the property of the selectivity-filter itself. For example, the selectivity-filter of a K^+^ channel possesses an energetic trap for the substrate K^+^ ions (as well as inhibitory ions), and the same selectivity-filter behaves as an energy barrier for other types of ions (Zhou *et al*. 2001). These detailed features of the energy landscape within the selectivity-filter are relevant only to the kinetics, but can be omitted from our thermodynamic consideration. Taken together, binding of an ion to one terminal site favors movement of ions inside the selectivity-filter towards the opposite terminal site. In the absence of ∆*Ψ*_M_, the final direction of the movement inside the filter would be determined by the transmembrane gradient of the substrate concentration. This is analogous to the movement of a two-way door, in which the side of stronger pressure wins. In fact, the chemical potential of the substrate, ∆*µ*(S) (≡*RT*·ln([S]_R_/[S]_L_), where the subscripts R and L stand for releasing and loading, respectively, drives the substrate movement, regardless of the charge(s) carried by the substrate. In this scenario, the electrostatic forces between the charges of the substrate ions are only a medium for transmitting the information about the chemical potential, but are not the bona fide driving force. In the presence of ∆*Ψ*_M_, the ∆*µ*-driven ion movement is either accelerated or slowed down (even reversed) by ∆*Ψ*_M_, depending on the direction and strength of ∆*Ψ*_M_ relative to the concentration gradient. Moreover, the average concentration of net charges to maintain ∆*Ψ*_M_ is low compared to the local concentration associated with a tightly bound ion to a membrane protein. Assuming a value of 100-mV for ∆*Ψ*_M_ and a 30-Å thickness (*d*) of the membrane, the net charge density (*Q*/*A *= ∆*Ψ*_M_*C*/*A* = *ε*_0_∆*Ψ*_M_/*d*, where *A* and *C* stand for surface area and electric capacity, respectively) on each side of the membrane is estimated as being only ~0.02 e_0_ per 1000*A*^2^ surface—equivalent to the cross section of a typical membrane protein. Therefore, binding of a single ion at one end of the selectivity-filter will strongly affect the electrical field within the selectivity-filter. In a real channel, however, such an effect is attenuated by differential binding of ions between the two opposite terminal sites. In this sense, ∆*µ*(S) is equivalent to an imaginary transmembrane electrostatic voltage, *V*_N_. This Nernst voltage is only applicable to a conduction pathway that connects the two spaces defining the ∆*µ*(S).

## Dry/wet two-state model of the gating process

**Fig. 2:**
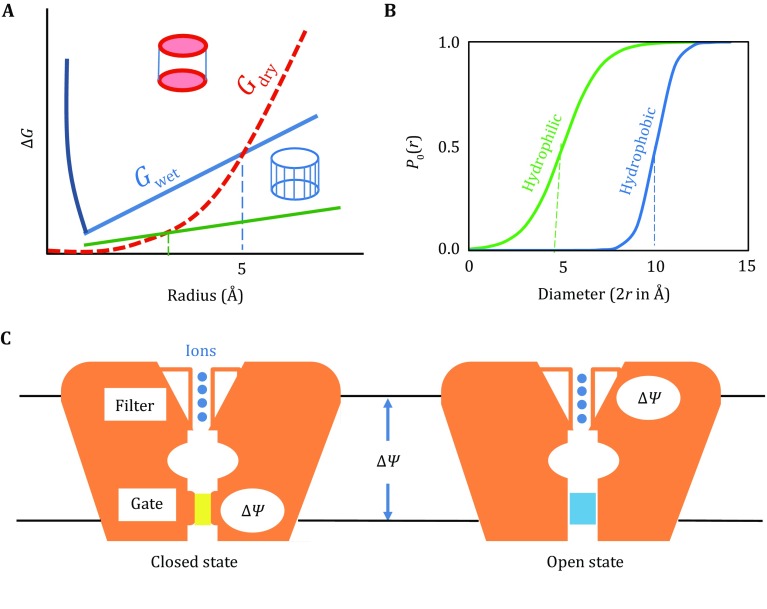
Dry/wet two-state model of the activation-gate. **A** Gibbs free energies of the dry (*red*, labeled as *G*_dry_) and wet (*blue*, *G*_wet_) states as functions of the radius of the activation-gate. Their crossing point corresponds to the critical radius (*r*_c_). Increasing the hydrophobicity of the gate decreases the slope of the *G*_wet_ curve (*green*), resulting in a reduced critical radius. **B** Open probability of the gate, *P*_o_(*r*) (≡[1 + exp((*G*_wet_ −*G*_dry_)/*RT*)]^−1^). Increasing the hydrophilicity of the gate shifts the *P*_o_ curve to a smaller *r*_c_. **C** Schematic diagram of the gating process. In the dry state (with a *yellow* vapor seal), ∆*Ψ*_M_ is loaded on the activation-gate; in contrast, in the wet state (with *cyan* water filling), ∆*Ψ*_M_ is loaded on the selectivity-filter

Thermodynamics of the gating process of the hydrophobic activation-gate has been described using a continuous-medium model (Anishkin *et al*. 2010; Anishkin and Sukharev 2004). In this simplified yet elegant two-state model, the activation-gate is represented by a cylindrical pore which has a hydrophobic inner surface. In the close state of the gate, the size (*i.e.*, radius) of the cylinder is small, and its pore is narrow and “dry”; in contrast, in the open state, the size of the cylinder increases, and its pore turns “wet.” More specifically, in the dry state, a vapor seal exists inside the cylinder and expels water and other polar solvent molecules as well as ions, and in the wet state, the hydrophobic cylinder becomes filled with aqueous solvent. Two conflicting energetic factors determine the state of the activation-gate cylinder (Fig. 2). On the one hand, the dry state maintains two aqueous-gas interfaces at the two ends of the vapor seal, and thus the dry state is associated with an energy term proportional to the area (or the radius square) of the cross section of the activation-gate. On the other hand, filling the hydrophobic activation-gate with aqueous solvent also consumes energy. This second energy term is proportional to the area of the wetted inner surface of the cylinder, and thus increases linearly with the radius of the pore cylinder. Therefore, whether the activation-gate is dry or wet depends on the radius of the activation-gate. Once the radius of the activation-gate becomes larger than the so-called “critical radius” of wetting (*r*_c_, approx. 5 Å (Anishkin *et al*. 2010)), the wet state of the activation-gate becomes less energetically costing than the dry state, the vapor seal breaks, and the cylinder pore is filled with polar molecules and ions. The open probability *P*_o_(*r*) of the activation-gate as a function of the gate radius is determined by the differential Gibbs energy, ∆*G*_dw_ (≡*G*_wet_ −*G*_dry_), according to the Boltzmann distribution:

1 $$P_{\text{o}} \left( r \right) \, = \, \left[ { 1+ { \exp }\left( {\Delta G_{\text{dw}} \left( r \right)/RT} \right)} \right]^{ - 1} .$$

At the critical radius, ∆*G*_dw_(*r*_c_) equals to zero, and the two states are evenly distributed. The *P*_o_(*r*) curve is likely to be steep around the critical radius, meaning that a small structural change may have a large effect on the open probability of the activation-gate. As discussed below, the structural changes associated with ∆*G*_dw_ are usually driven by other energy sources (*e.g.*, the differential conformational energy of the activation-gate, ∆*G*_gating_). The concept that a small structural adjustment in the activation-gate region is able to trigger an instantaneous state transition of the activation-gate is consistent with rapid activation of ion channels (Bagneris *et al*. 2015). This two-state mechanism has been convincingly applied to mechanosensitive channels to explain their gating mechanism (Anishkin *et al*. 2010).

During gate opening, the four pore-lining S6 helices either physically move away from each other (thus increasing the “radius” of the activation-gate) or chemically increase the hydrophilicity of the activation-gate (thus reducing both the energy cost of wetting and *r*_c_). In any case, a necessary condition for the activation-gate to supply the selectivity-filter with a continuous ion flow is to have the cross section of the gate larger than the size of hydrated ions. In experimentally determined channel structures (*e.g.*, in Na_V_Ms/(PDB IDs: 3ZJZ and 4F4L) (Bagneris *et al*. 2013; McCusker *et al*. 2012) both radially outward bending and rotation of S6 helices are observed, resulting in dilation of the activation-gate and increase of the hydrophilicity of the gate pore. These conformational changes are based on a pivot at the middle point of S6 (called gating-hinge, *e.g.*, at the residue T206 in Na_V_Ms), and were considered as structural evidence to support the original “hinge motion hypothesis” on channel gating (Zarrabi *et al*. 2010). However, this hypothesis alone may lead to a model of continuous gating. In contrast, combining it with the two-state model better accounts for the common observation of one-or-none conductance of a single channel.

How is the state change of the activation-gate translated into instantaneous channel opening? In the open state of the channel, the movement of the substrate ions inside the channel is driven by ∆*Ψ*′. The central pore can be considered as an electric circuit of two resistors of the activation-gate and selectivity-filter. The voltage-drop on each part of the circuit is proportional to its resistance (*i.e*., reciprocal of the conductance) relative to the overall resistance of the series-connected circuit. In the dry state, the activation-gate has a very large resistance, practically approaching to infinity. Thus, the voltage-drop applied to the selectivity-filter is zero, because the activation-gate draws all of ∆*Ψ*_M_. In this case, an induced electric dipole is formed by both cations trapped inside the selectivity-filter and anions attracted locally on the positive side of ∆*Ψ*_M_. While this induced dipole cancels the electrical field of ∆*Ψ*_M_ inside the selectivity-filter, it also strengthens the field across the activation-gate. Therefore, the electric current passing the selectivity-filter is zero, and consequently the channel is closed. Importantly, this closure functions only in the sense of electric conduction, because the physical size of the activation-gate may remain sufficiently large to hold a hydrated ion (though unable to do so because of the vapor seal). In contrast, in the wet state, the activation-gate is filled with solvent exhibiting a high dielectric constant. Water molecules inside the activation-gate become polarized by the external electrical field of ∆*Ψ*_M_, and their induced dipoles (partially) cancel the external electrical field. Consequently, the total electrical field inside the activation-gate approaches to zero, and nearly the entire ∆*Ψ*_M_ is focused on the selectivity-filter, driving the ion movement. Because of this electric nature of the gating process, a small structural change around the critical radius of *P*_o_(*r*) instantaneously reshapes the electrostatic field of ∆*Ψ*_M_, and thus generates a quick response of the channel current to the activation cues that regulate the pore size.

## Voltage sensor

### Sliding versus rocking models

In the voltage-gated, tetrameric 6TM, ion channels, the voltage sensor (VS) is comprised of transmembrane helices S1–S4. ∆*Ψ*_M_ determines the conformation of the VS domain, and thus the change of ∆*Ψ*_M_ (*i.e.*, ∆∆*Ψ*_M_) is responsible for the conformational change. In response to ∆∆*Ψ*_M_, the VS domain regulates the opening of the activation-gate. Homologous functional VS domains are also found in non-channel membrane proteins (Sakata *et al*. 2016), suggesting that these proteins utilize a common voltage-sensing mechanism conferred by the VS domains. For most known VGICs, the open probability (*P*_o_(*V*)) of the activation-gate increases as the ∆*Ψ*_M_ is depolarized from its resting value. Thus, this type of channels is specifically called depolarization-activated channels. In contrast, for hyperpolarization-activation cyclic nucleotide-gated (HCN) channels, the activation-gate is opened when the ∆*Ψ*_M_ becomes more polarized (Novella Romanelli *et al*. 2016). This latter type of channels is responsible for restricting the resting ∆*Ψ*_M_ and for pace-making in cardiac cells (Jackson *et al*. 2007). Although the VS domains from the two types of channels show distinct effects in response to membrane polarization, they are homologous in their primary sequences and share similar overall 3D structures (Jackson *et al*. 2007; Lee and MacKinnon 2017). More specifically, VS domains from both types of voltage-gated channels contain a conserved, highly positively charged S4 helix, part of which assumes a 3_10_-helix conformation (Long *et al*. 2005). Because of this 3_10_ conformation, 4–6 basic residues residing on S4 (referred to as R0–R5) are aligned on the same side of the helix surface. For example, in Na_V_ channels, four conserved Arg residues (R1–R4) are observed in S4 (Bagneris *et al*. 2015). These basic residues are believed to contribute to the gating-charges of the VS domain (Armstrong and Bezanilla 1973; Bagneris *et al*. 2015; Islas and Sigworth 2001; Mannuzzu *et al*. 1996; Stuhmer *et al*. 1989).

Unlike a selectivity-filter, the VS domain does not sense the Nernst potential. In fact, in the time scale of conformational changes of a VS domain, the electric field of ions responsible for the Nernst potential is effectively screened out by the surrounding solution including counter ions; however, the same argument would not be true for the microscopic environment of a selective-filter. Thus, a leak-free VS domain changes its conformation in response only to ∆∆*Ψ*_M_, but not to *V*_N_ (Benedek and Villars 2000). Upon depolarization of the membrane potential, the VS domain undergoes a rather large conformational transition, translocating positive gating-charges from the cytosolic side to the extracellular side of the ∆*Ψ*_M_. The precise mechanism of the charge translocation remains under debate. A currently favored hypothesis is that the positively charged S4 undergoes a large outward sliding movement relative to the membrane (Catterall 2010). This sliding mechanism is consistent with results from disulfide-bond formation experiments, which trapped a few putative intermediate states during activation of the VS domain (DeCaen *et al*. 2009), and with the high degree of conservation of the R1–R4 residues aligning on the S4 surface. Nevertheless, direct structure evidence for such sliding hypothesis has not been reported thus far. An alternative charge-translocating mechanism is the rocking-like two-state model similar to that of many transporters. In such a rocking model, the conformation of the VS domain changes between an outward-facing conformation (C_out_) and an inward-facing conformation (C_in_, where the in/out orientation is referred to channels on the plasma membrane) (Chanda *et al*. 2005; Kintzer and Stroud 2017). This rocking mechanism is supported by a fluorescence experiment showing that the S4 helix does not move significantly relative to the membrane lipid bilayer (Chanda *et al*. 2005). In addition, certain histidine substitutions of arginine residues in S4 are shown to convert the VS domain into a proton transporter, suggesting that a voltage sensor and a transporter mechanistically function in a similar manner (Starace and Bezanilla 2001). It should be noted that both the sliding model and the rocking model appear to agree with results of the studies conducted to investigate chemical reactivity of cysteine mutations (Yang and Horn 1995). Other studies using voltage-clamp fluorometry (Cha and Bezanilla 1997; Mannuzzu *et al*. 1996) indicated that upon the change of membrane potential, a large conformational change within VS results in the translocation of gating-charges (Catterall 2010). A major question concerning the difference between the two models is whether the conformational change is via sliding of S4 relative to the membrane (Catterall 2010; Ruta *et al*. 2005) or a rocking-like motion (Yellen 2002).

**Fig. 3:**
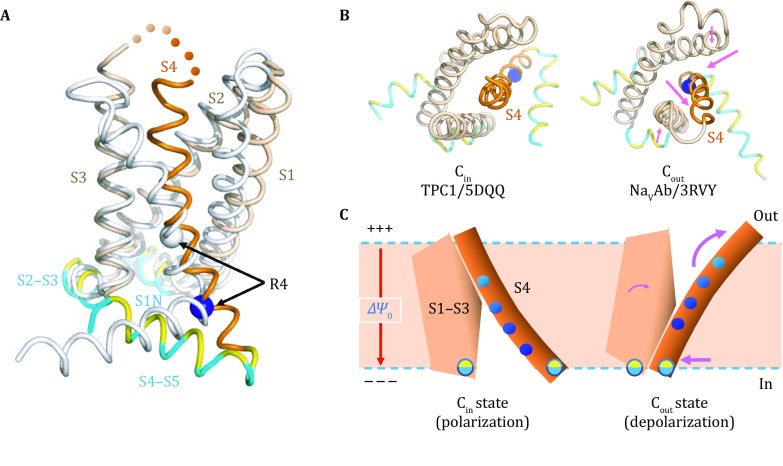
Sliding-rocking two-state model of the voltage-sensor domain. **A** Superposition of VS domains from TPC1/5DQQ (C_in_ state) and Na_V_Ab/3RVY (C_out_). The C_in_ structure is colored in *wheat color* (S1, S2, and S3), *orange* (S4), and *cyan-yellow mixed* (amphipathic helices), and the C_out_ structure is colored in *gray* for clarity. Positions of one of the four conserved basic residues, R4, in the two structures are marked with *blue* and *gray spheres*, indicating a two-turn sliding movement of S4 relative to the remaining VS domain. S2 and S3 as well as the amphipathic linker helix (S2–S3) are superimposed well. **B** Extracellular view of the VS domains shown in panel (**A**). **C** Schematic diagram of the sliding-rocking model. In the polarization state, the VS domain has an inward-facing conformation (C_in_). Upon depolarization, the outward-facing conformation (C_out_) becomes energetically favored. The voltage change is sensed by positively charged residues in S4, R1–R4 marked as spheres from *light to dark blue*. Movements of structural elements are indicated by *magenta arrows*. During the conformational change, the cytosolic ends of S1–S4 are anchored to the cytosolic surface of the membrane by amphipathic helices (depicted as *yellow-cyan circles*)

Based on currently available data, we propose an alternative putative mechanism, called sliding-rocking two-state model (Fig. 3): a VS domain uses a hybrid mechanism of the above two models. In this combined model, the sliding of S4 is relative to a polar slot formed by S1–S3, but not to the membrane per se, being accompanied by a rocking motion between the two parts of VS. In the research field of transporters, a similar mechanism is called an “elevator car” mechanism (McCoy *et al*. 2016). During the sliding-rocking movement of the VS domain, two crevices are created alternatingly on the two sides of the membrane, resulting in drastically reshaping of the electrical field of ∆*Ψ*_M_ inside the VS domain and at the same time translocating the gating-charges relative to the electrical field.

### Structural changes between the two states

Currently, the only VS domain for which the structures of both inactive and active states have been reported was isolated from a voltage-sensing phosphatase from *Ciona intestinalis* (Ci-VSD) (Li *et al*. 2014). As the registration of amino acid sequences (especially that of S4) into weak electron densities is ambiguous, the presumed “inactive” C_in_ structural model of Ci-VSD remains uncertain. Moreover, whereas the experimental C_out_ structures of VS domains have been repeatedly reported [*e.g.*, Kv1.2/2A79 (Long *et al*. 2005) and Na_V_Ab/3RVY (Payandeh *et al*. 2011)], structural information of the C_in_ state is limited to that of TPC1/(5DQQ and 5E1 J) from *Arabidopsis thaliana* (Guo *et al*. 2016; Kintzer and Stroud 2016). The dominance of C_out_ states in reported structural studies may be partially explained by the lack of ∆*Ψ*_M_ under experimental conditions for the structural data reported.

In both C_in_ and C_out_ structures of VS domains (Fig. 3A), the S4 helix is slightly curved, with its positively charged, convex surface facing the remaining part of the VS domain. The R1–R4 residues are located in the 5th–2nd turns (counted from the C-terminal end of S4), respectively. In the *in vivo* membrane environment, the C-terminal end of S4 is likely anchored to the cytosolic surface of the membrane by the following amphipathic S4–S5 helix. Moreover, in both C_in_ and C_out_ structures, S1, S2, and S3 form a transmembrane hydrophilic slot, with an “N”-shaped topology for the peptide chain. This slot provides the positively charged S4 surface with a low energy-barrier sliding track for translocation of the gating-charges. The overall hydrophilic property of their interface is further supported by the observation that a single-point mutation at R1 created a proton-wire across the TM region of the VS domain (Starace and Bezanilla 2001). In the middle of the N-shaped slot is the so-called gating-charge-transfer center (Tao *et al*. 2010), which contains both a conserved negatively charged residue partially neutralizing positive charges from S4 and a conserved hydrophobic residue acting as a seal to prevent charge leakage.

Structural comparison of the two states provides further support to the sliding-rocking model. In the C_out_ structure of Na_V_Ab/3RVY, the S1–S2 loop (*i.e.*, the loop connecting S1 and S2) separates from the extracellular end of S4, while the four helices S1–S4 are bundled together within the intracellular region (Fig. 3). The separation between S1–S2 and S4 creates an aqueous crevice (~10-Å or deeper) penetrating from the extracellular space into the TM region. Three of the positively charged residues in S4 (R99^(R1)^, R102^(R2)^, and R105^(R3)^ of Na_V_Ab) face the interior of this crevice, and are thus all located on the positive side of ∆*Ψ*_M_. In comparison, in the C_in_ structure of TPC1/5DQQ, the S1–S4 helices of the functional VS-II domain become bundled together in the extracellular region; the C-terminal half of S4 separates from the S1–S3 helix bundle in the intracellular region. This structural rearrangement eliminates the extracellular crevice, but instead creates an aqueous crevice (deeper than 10-Å) penetrating from the cytosolic side into the TM region. Three of the positively charged residues in S4 (R537^(R2)^, R540^(R3)^, and R543^(R4)^ of TPC1) face the interior of the intracellular crevice, and are thus all located on the negative side of ∆*Ψ*_M_. The rearrangement of S4 relative to S1–S3 during the C_in_-to-C_out_ (I–O) transition results in a two-turn sliding towards the extracellular direction (Fig. 3A) (She *et al*. 2018), in agreement with the observation from the disulfide-bonding experiment (DeCaen *et al*. 2009). This sliding movement of S4 requires local conformational change in the S3-S4 linker. In fact, this linker region is invisible in several VS crystal structures, indicating high flexibility, and varies in both amino acid sequence and length between species. By probing with a variety of neurotoxins, the extracellular S3–S4 loops in several VGICs are shown to assume different conformations between the resting and activated states (Catterall 2010). With increasingly larger truncation in this loop, mutants of the Shaker *K*_V_ channel showed slower activation rates and more right-shifts of the activation curve, indicating that the channel becomes harder to open (Gonzalez *et al*. 2000). In comparison, the linker between the C-terminal end of S4 helix and the S4–S5 amphipathic helix is always well defined in the crystal structures, although the angle between the two helices changes from ~35° in C_in_ to ~80° in C_out_ (Fig. 3A). It is important to re-emphasize that, during conformational transitions between C_in_ and C_out_, the C-terminal end of S4 likely remains anchored to the intracellular surface of the membrane by the S4–S5 amphipathic helix. Similarly, in a number of ion channels, the N-terminal end of S1 is also found to be linked to a short amphipathic helix, S1 N, and S2 and S3 are connected by a short amphipathic helix S2–S3. Together, these amphipathic anchors on the intracellular surface impose strong restraints on possible conformational changes of the VS domain. For the experimentally observed conformational change to occur in a membrane environment while keeping the anchoring in effect, the two parts of VS, *i.e*., S4 and the S1–S3 bundle, are mostly likely to rock relatively to each other as well as to the membrane (Fig. 3C). Consequently, the structural difference between C_in_ and C_out_ results in translocation of the gating-charges relative to the focused electrical field of ∆*Ψ*_M_, supporting the sliding-rocking mechanism of the VS domain.

As a side note, the *A. thaliana* channel TPC1 is interesting in several aspects, besides providing structural information on the C_in_ state. TPC1 is composed of a homodimer, and each of the two subunits contains two VS-like domains (VS-I and VS-II). Of the four putative VS domains, only two diagonally arranged VS-II domains are functional in voltage sensing. Activation of the channel requires both the voltage sensing by the two VS-II domains and Ca^2+^ binding to cytosolic domains which are attached to the pseudo-VS-I domains (Guo *et al*. 2016). Furthermore, binding of Ca^2+^ on the extracellular/vesicle-lumen side of the VS-II domains in the C_in_ state inhibits the channel opening (Guo *et al*. 2016). This inhibitory effect is probably a consequence of locking together the S1–S2 and S3–S4 loops, and also a result of exerting an extra inward electrostatic force on the VS-II domain, both stabilizing the C_in_ state.

### Gating-charges

The basic residues residing along the S4 helix are proved to be critical for voltage sensing (Catterall 2000, 2010). The inward electrostatic forces that are exerted on these basic residues increase with polarization of the negative-inside ∆*Ψ*_M_. Similarly, other charged residues residing on S2 and S3 (mostly being conserved acidic residues) also sense ∆∆*Ψ*_M_. These changes of electrostatic forces are responsible for the relative movements between S4 and the S1–S3 bundle, switching the VS domain between C_out_ and C_in_. Whereas the conserved positively charged residues concentrated in S4, namely R1–R4, are likely to be the main contributors to the overall gating-charges, all charged residues contribute to the gating-charges with varied weights (Online Appendix) (Islas and Sigworth 2001). More specifically, whether a charged residue makes a significant contribution to the overall gating-charges ultimately depends on its positional shift relative to the electrical field of ∆*Ψ*_M_ during the conformational transition of the VS domain.

Another important force exerted on membrane proteins is the hydrophobic mismatch (Phillips *et al*. 2009), which originates from a positional mismatch between the hydrophobic surface of the membrane protein and the surrounding lipid bilayer. Like electrostatic forces, the forces generated by hydrophobic mismatches also change in magnitude and even direction in response to ∆∆*Ψ*_M_, keeping the protein to reside inside the membrane bilayer. The relationship between the electrostatic force and hydrophobic mismatch can be analogous to the interaction between a floating boat and its surrounding water. Just as the gravitational force pulling the boat downwards is balanced by the buoyancy force that keeps the boat afloat, the C_in_ state of a VS domain is maintained by the balance between forces of hydrophobic mismatch and the inward electrostatic forces exerted by ∆*Ψ*_M_ on each and every positively charged residue (as well as the outward electrostatic forces on negatively charged residues). Upon ∆*Ψ*_M_-depolarization, the inward (outward) electrostatic forces exerted on basic (acidic) residues are reduced; the mechanical balance of the C_in_ state is broken; distinct mechanical torques are generated on different helices of the VS domain, promoting their relative movements. As a result, the overall conformation of the VS domain changes, and in the process a new state, C_out_, equilibrating between electrostatic and hydrophobic forces is established. Importantly, during the state transition, the tilting angle of S4 relative to the membrane normal is likely to change, a process that is accompanied by distinct patterns of charge exposure. A similar ∆*Ψ*_M_-driven conformational change has been proposed for proton motive force (PMF)-driven transporters, referred to as membrane potential-driving hypothesis (Zhang *et al*. 2015, 2017a), which is an extension and realization in transmembrane transport of the chemiosmotic theory proposed by Peter Mitchell. However, these two types of membrane proteins exhibit one major difference: in PMF-driven transporters, it is the change of electric charge (associated with protonation and deprotonation) that drives the transport cycle. In contrast, in the VS domain, it is the change of membrane potential that drives the conformational change during voltage sensing.

In addition to the alternating patterns of gating-charge exposure, each of the C_in_ and C_out_ states seems to be stabilized by a distinct group of interaction within the VS domain, including salt-bridge bonds. For instance, in the C_out_ structure of Na_V_Ab/3RVY, residues E59^S2^, R63^S2^, D80^S3^, and R108^S4(R4)^ form a charge cluster in the cytosolic half of the VS domain. It can be envisioned that, in the C_in_ state, these interactions are lost; instead, new interactions are formed in the extracellular half of the VS domain, including a salt-bridge bond between E32^S1^ and R99^S4(R1)^. Similarly, in the C_in_ structure of TPC1/5DQQ, E511^S3^–R531^S4(R0)^ and D500^S3^–R540^S4(R3)^ pairs are observed to form two salt-bridge bonds. These distinct clusters of charge pairs probably contribute to the two-state mechanism of VS by stabilizing either the C_in_ or C_out_ state (Papazian *et al*. 1995). The role played by paired charges in stabilizing of the VS domain appears to be similar to that of A-like motifs conserved in MFS transporters, where these motifs stabilize their transporters in the two terminal states of a functional cycle (Zhang *et al*. 2015). Point mutations in such clusters, if breaking the charge pairs, will result in reduced stability of either the C_in_ or C_out_ state, skewing the two-state transition process. For example, mutations at the basic residues in the cytosolic half of S4 cause a right shift of the *I*–*V* curve of the rat Na^+^ channel II, indicating destabilization of the C_out_ state. In contrast, mutations in the extracellular half of S4 result in a left shift (Stuhmer *et al*. 1989). Similarly, mutations at acidic residues that are involved in charge-pairing often show drastic effects. For example, the D60K mutation in a cytosolic charge cluster of NaChBac shifts the *I*–*V* curve 75-mV towards depolarizing potentials (*i.e*., more difficult to be activated) (Zhao *et al*. 2004), presumably by over-stabilizing the C_in_ state via electrostatic interaction with ∆*Ψ*_M_ and by destabilizing the C_out_ state via disrupting charge-pairing. In addition, substituting R1 with a small side-chain residue resulted in formation of a cation channel running through the VS domain in its C_in_ conformation (Tombola *et al*. 2005b), presumably by breaking the seal formed by the charge paring in the C_in_ state. Moreover, folding defects from mutations that neutralize basic residues in S4 can be suppressed by neutralization of acidic residues in either S2 or S3 (Papazian *et al*. 1995). Of particular interest are insights gained from the charge-reversal mutations in the *Drosophila* Shaker *K*_V_ channel (Papazian *et al*. 1995; Tiwari-Woodruff *et al*. 2000), in which the paired, charged residues in the VS domains were mutually switched yet the mutants maintained the channel activity. Taken together, while unpaired charges are more likely to contribute to the gating-charges, the paired charges stabilize the terminal states, and alternating stabilization of both states is essential for proper functioning of the VS domain.

### Cooperativity between voltage-sensing domains

As discussed above, the four VS domains encircle the central pore of the channel. An important question concerning the function of VGICs is whether the four VS domains function cooperatively. If so, a further question is what mechanism for cooperation can be envisaged that plausibly explains the observed voltage-activation relationship.

Upon depolarization and I–O transition, the number of positively charged residues exposed to the cytosol decreases, while the number of positively charged residues exposed to the extracellular space increases. Thus, for each voltage sensor, an efflux of net positive charges occurs across the focused electrical field of ∆*Ψ*_M_ (or of the experimentally applied external voltage, *V*). At any given external voltage, the charge movement related to the I–O transition would be associated with an energy term *V*∆*Q*. Here, we re-define ∆*Q* as the gating-charges of a single VS “unit.” Such a unit is either a fully cooperated ion channel (with four VS domains) or one independent VS domain. As noted above, ∆*Q* is contributed by all charged groups from the unit. Previously, the total gating-charges of the skeleton muscle Na^+^ channel were estimated to be 3 per VS domain (or ~12 per channel) (Hirschberg *et al*. 1995), and for the KvAP channel, 2.4 per VS domain (Schmidt *et al*. 2009). Let the gating-charges per VS domain be 2.5 e_0_, ∆∆*Ψ*_*M*_ be 50 mV, and the dielectric constant (*ε*) for the protein be 2. In the focused electrostatic field associated with the VS crevice (see Supplementary Fig. S2), the average electrostatic force that drives the conformational change of each VS domain is estimated to be ~5 pN, which is fairly strong for a molecular event.

The Gibbs free energy of the I–O transition of the VS domain, ∆*G*_VS_, can be described as

2 $$\Delta G_{\text{VS}} \left( V \right) \equiv G_{\text{out}} \left( V \right) \, {-}G_{\text{in}} \left( V \right) \, = \, \Delta G^{0} {-}V\Delta Q,$$

3 $$\Delta \Delta G_{\text{VS}} \equiv \Delta G_{\text{VS}} \left( {V_{1} } \right) \, {-} \, \Delta G_{\text{VS}} \left( {V_{0} } \right) \, = \, \Delta V\Delta Q,$$

where *G*_out_ and *G*_in_ are Gibbs energies corresponding to the C_out_ and C_in_ conformations at a given voltage, ∆*G*^0^ represents their differential conformational energy at *V* = 0 mV, and *V*_0_ and *V*_1_ are the resting and activation voltages, respectively. This description of ∆*G*_VS_ mirrors that of membrane tension-induced conformational change in mechanosensitive channels, with *V* and ∆*Q* equivalent to membrane tension (*σ*) and change of the cross-section area (∆*A*), respectively (Zhang *et al*. 2016). Here, we follow the convention of thermodynamics according to which the negative ∆*G* is associated with an exergonic process. For most known VGICs, ∆*G*^0^ < 0, indicating that the C_out_ state is more stable at *V* = 0 mV (one exception is the above-mentioned Ci-VSD, which assumes a C_in_ state in the absence of membrane potential and has a *V*_1/2_ of +60 mV (Li *et al*. 2014)). Since ∆*G*^0^ can be modulated by other mechanisms (*e.g.*, ligand binding), a voltage-gated channel is often regulated by factors in addition to ∆*Ψ*_M_. Moreover, to open the activation-gate, VS usually acquires energy from the activation cues, thus coupling ∆∆*G*_VS_ with ∆*G*_gating_ (which includes but is not limited to ∆*G*_dw_). Because of this coupling, mutations at the activation-gate (*e.g.*, those reported in Zhao *et al.* 2004) may dramatically shift the *I*–*V* curve by affecting ∆*G*^0^ in Eq. 2. Furthermore, in mammalian Na_V_ channels, the four homologous VS domains display distinct activation kinetics (Chanda and Bezanilla 2002). In the structure of NavPaS/5X0M, the gating-charges in different S4 helices assume different positions relative to the S1–S3 bundle (Shen *et al*. 2017). These observations suggest that different VS domains of the same channel may have distinct ∆*Q* and ∆*G*^0^ values.

For the purpose of our theoretical analysis, we assume that (1) the activation-gate is controlled by *n* identical, fully independent VS units; and (2) in order to be activated by ∆∆*Ψ*_M_, the channel opening requires all *n* copies of the units to function collectively (Schmidt *et al*. 2009). Therefore, the open probability, *P*_o_(*V*), of a depolarization-activated channel can be described as a Boltzmann distribution

4 $$P_{0} \left( V \right) = \left[ {\frac{1}{{1 + \exp \left( {\frac{{\Delta G_{\text{VS}} }}{RT}} \right)}}} \right]^{n} .$$

Thus,

5 $$P_{0}^{'} \left( V \right) \equiv \frac{{{\text{d}}P_{0} }}{{{\text{d}}V}} = \frac{n\Delta Q}{RT} \cdot P_{0} \left( V \right) \cdot \frac{1}{{1 + { \exp }\left( {\frac{{{-}\;\Delta G_{\text{VS}} }}{RT}} \right)}},$$

6 $$\frac{n\Delta Q}{F} = \frac{2RT}{F} \cdot P_{0}^{'} \left( {V_{1/2} } \right) \cdot \frac{{\sqrt[n]{2}}}{{\sqrt[n]{2} - 1}} \approx 50\;{\text{mV}} \cdot P_{0}^{'} \left( {V_{1/2} } \right) \cdot \frac{{\sqrt[n]{2}}}{{\sqrt[n]{2} - 1}},$$

7 $$\Delta G^{0} = V_{1/2 } \cdot \Delta Q + RT \cdot \ln (\sqrt[n]{2} - 1),$$

where *V*_1/2_ is defined as the voltage at which *P*_o_(*V*) = 1/2 (Zarrabi *et al*. 2010). For a hyperpolarization-activated channel, replace the ∆*G*_VS_ with −∆*G*_VS_. Regarding these equations, a few points are worth discussion:

1. *P*_o_(*V*), *P*_o_’(*V*), as well as *V*_1/2_ are experimentally measurable. The total gating-charges (*n*∆*Q*/*F*, in e_0_ unit) and ∆*G*^0^ can be derived using Eqs. 6 and 7.
2. The derived results depend on the model being used (*e.g*., whether *n* equals 1 or 4). Here, the independency coefficient, *n*, is reciprocally related to the Hill coefficient (see §11 in Hill 1985). Whereas the traditional cooperative model of VGICs is equivalent to *n* = 1 (Zarrabi *et al*. 2010), full independence between the four VS domains implies that *n* = 4 (Schmidt *et al*. 2009).
3. The larger are the total gating-charges *n*∆*Q*/*F*, the steeper is the *P*_o_(*V*) curve at *V*_1/2_ (*i.e.*, a larger *P*_o_’(*V*_1/2_)), corresponding to a more precise control of ∆*Ψ*_M_ over the state transition of the VS unit.
4. For depolarization-activated channels, *V*_1/2_ is more positive than the resting ∆*Ψ*_M_; for hyperpolarization-activated channels, *V*_1/2_ is more negative than the resting ∆*Ψ*_M_. Because of the linear relationship between ∆*G*^0^ and *V*_1/2_, any cellular factors that modulate ∆*G*^0^ will affect *V*_1/2_ too. For example, cAMP binding to the cytosolic domain of the hyperpolarization-activated HCN channel shifts *V*_1/2_ to more positive values, thus easier to open the channel (Stieber *et al*. 2003).

For a given experimentally determined *P*_o_’(*V*_1/2_), the total gating-charges predicted based on the model of fully independent VS domains (*i.e.*, *n* = 4) would be approx. three times to that estimated based on fully cooperative gating (*i.e.*, *n* = 1). Therefore, it seems that the traditional, fully cooperative model is in better agreement with the structural analysis on available potential gating-charges than the fully independent model. Nevertheless, it is still possible that the four VS domains function in a partially cooperative manner (*i.e.*, 1 < *n* < 4). In case of full cooperativity, activation occurring in one VS domain will stimulate activation of other VS domains, through a currently unknown mechanism. If domain swapping were the cause of cooperativity, a non-swapped channel would be more likely to show full independency among its four VS domain. However, non-swapped HCN channels show even larger gating-charges (~5 *e*_0_ per VS domain, estimated based on *n* = 1 (Wainger *et al*. 2001)) than the typical (~2.5 *e*_0_) gating-charges in swapped VGICs. This observation strongly suggests that it is highly unlikely for the four VS domains in a HCN channel to act in a fully independent manner, as it would require ~15-*e*_0_ gating-charges per VS domain, a prediction unsupported by structural data.

On the basis of the above discussion, we propose a mechanistic model for the cooperativity in a canonical depolarization-activated ion channel. Since all four VS domains are linked to the activation-gate, their ∆∆*G*_VS_ terms are coupled with each other through ∆*G*_gating_ of the activation-gate. On the one hand, in the absence of compression force from the VS domains, the central pore of the channel is presumably present in its open state (*i.e.*, ∆*G*_gating_ ≡*G*_open_ − *G*_close_, < 0). Such an open state is probably stabilized by the *in vivo* membrane environment, and thus has not been observed in most of the reported crystal structures. On the other hand, the closed state of the central pore is maintained by compression forces from all four VS domains in their C_in_ state. Therefore, it appears that being in the C_in_ state for one of the four VS domains is a sufficient (but not necessary) condition for keeping the activation-gate close. In other words, being in the C_out_ state for all four VS domains is a necessary (but not sufficient) condition for keeping the activation-gate open. In energetic terms, under the condition of polarized ∆*Ψ*_M_, ∆*G*_gating_ of the activation-gate is canceled out by ∆*G*_VS_ contributed by the surrounding VS domains. Upon the I–O transition of one VS domain, the compression on the central pore is partially removed. Consequently, the force to maintain the closed state of the activation-gate is likely reshuffled among the remaining VS domains that are still in contact with the activation-gate. As a result, these remaining VM domains become overloaded, because they themselves are also in the margin of *V*_1/2_. This increased force, which originated from the negative ∆*G*_gating_ and is shared by the VS domains remaining in C_in_ states, will facilitate further I–O transition in other VS domains, increasing their probability to be activated (with a decreased ∆*G*^0^). In this domino manner, a cooperative activation of all four VS domains as well as the central pore is realized. Therefore, on the basis of the charged residues available in the VS domain, we propose that the four encircling VS domains in the tetrameric VGIC use a cooperative mechanism to activate the central pore.

### Coupling between the VS domains and the central pore

The voltage-sensing mechanisms are similar in both depolarization- and hyperpolarization-activated channels, performing the same I–O transition upon ∆*Ψ*_M_-depolarization. However, for the VS domain of a depolarization-activated channel, C_in_ corresponds to the resting state of the channel, and C_out_ corresponds to the activated state; for hyperpolarization-activated channels, the situation is reversed. Thus, for depolarization-activated channels the I–O transition is associated with the gate opening, whereas for hyperpolarization-activated channels, the same I–O transition is associated with the gate closing. In either case, part of the activation energy from the VS domain is coupled to the activation-gate, either removing energy barrier or compensating the energy cost of gate opening.

The question then arises as to how the VS domains communicate with the activation-gate. Two types of packing, swapped and non-swapped, have been found between the VS and pore-domains. While the swapped assembly is more commonly observed, the non-swapped assemblies are currently only found in the two crystal structures of Eag1/5K7L (Whicher and MacKinnon 2016) and HCN1/5U6O (Lee and MacKinnon 2017). We first discuss the swapped assembly, in which the VS domain from each subunit packs sequentially against the pore domain of its neighboring subunit. The VS domain contacts the central pore through multiple patches of interaction, including an inter-subunit contact between the S1–S2 loop and C-terminus of S5’ (where the prime indicates being from a neighboring subunit); an extensive inter-subunit contact between anti-paralleled S4 and S5’; and an intra-subunit, VS-pore connection through the amphipathic linker helix, S4-S5 (Long *et al*. 2005; Payandeh *et al*. 2011). During the conformational transition of the VS domain, these multiple contacts change their relative arrangement, transducing ∆*G*_VS_ from the VS domain to the central pore. For example, while in the *C*_in_ state of the structure of Na_V_Ab, a salt-bridge bond is presumably formed between K35^S1^ and E154^S5’^, this bond is lost in the C_out_ structure (3RVY) (Payandeh *et al*. 2011). Disruption of these inter- and intra-subunit interactions is likely to perturb the communication between the VS domains and the central pore. Intriguingly, a number of disease-related mutations in human Na_V_ channels have been mapped to the interface between S4 and S5’ (Shen *et al*. 2017). Experimentally, mutations in the S1–S2 loop region were found to affect the activation rate (Stieber *et al*. 2003). In addition, the signal transduction and energy coupling are believed to be relayed by the amphipathic S4–S5 helix, which connects S4 of the VS domain to S5 of the pore domain. This inter-domain connection is, however, likely to be only part of the function of the S4–S5 helix. In general, amphipathic helices play important roles in restricting conformational changes of transmembrane helices (Zhang *et al*. 2017b). As mentioned above, in 6TM channels, the S4–S5 helix anchors the cytosolic termini of both helices S4 and S5 to the intracellular surface of the lipid bilayer, converting “vertical” forces (*e.g.*, those associated with ∆*Ψ*_M_ and hydrophobic mismatch) perpendicular to the membrane to “horizontal” movements of the helix termini parallel to the membrane surface. Therefore, this anchoring effect of the S4–S5 helix is an important component of the sliding-rocking model of VS. More importantly, the horizontal movement of the C-terminus of S4 adjusts the size of a constricting ring formed by the four S4–S5 amphipathic helices surrounding the activation-gate, and thus controls the gating process. Firstly, during the O–I transition, the movements of both S4 and S4–S5 helices result in a change of the angle between them, and presumably tightens the constricting ring of the activation-gate, thus forcing the channel to close (Payandeh *et al*. 2011). The communication between VS and the central pore is reciprocal. For instance, preventing the central pore from entering its close state by using a blocker molecule has been shown to significantly slower the rate of the O–I transition of VS domains in the Shaker *K*_V_ channel (Tombola *et al*. 2005b). Secondly, in its C_out_ state, the VS domain loosens the constricting ring and releases the compression on the activation-gate, thus allowing the gate to open. Similar mechanism of coupling between S4 and the pore domain has been proposed based on the structural analysis on the EeNa_V_1.4 channel (Yan *et al*. 2017). Non-swapped, depolarization-activated channels (*e.g*., Eag1) are likely to use a similar mechanism to gate the channel, albeit in this case the interactions are all intra-subunit. Moreover, in the 3D structure of hyperpolarization-activated channel HCN1/5U6O, both the amphipathic helix S4–S5 and the constricting ring are absent. Instead, S4 and S5 are connected through a short loop. For such cases we hypothesize that, during the O–I transition (*i.e.*, activation by hyperpolarization), movement of S4 displaces S5 from the activation-gate and removes constrain of S5 on S6, thus opening the gate. In all cases, the movement of the cytosolic terminus of S4 relative to the central pore seems to function as a trigger to activate the gate of the central pore.

The VS domain may lose its coupling with the pore domain under certain *in vitro* conditions, as exemplified in the crystal structures of Na_V_Ab/3RVY and Na_V_Rh/4DXW (Zhang *et al*. 2012). In these structures, the VS domain appears in the active C_out_ state, but the activation-gate remains closed. This apparent inconsistency presumably results from the response of certain channels to the loss of the native membrane environment (including both ∆*Ψ*_M_ and proper hydrophobic interaction). In addition, in the Eag1 channel (a depolarization-activated non-swapped channel), the VS domain and pore domain can be decoupled by calmodulin (Whicher and MacKinnon 2016). Binding of calmodulin to the intracellular domains directly compresses the C-termini of S6 helices, and thus the gate becomes non-responsive to voltage-controlled S4 movement. Interestingly, certain Pro-substitution mutations in the pore-lining helix S6 of the depolarization-activated tetrameric Na^+^ channel, NaChBac, result in hyperpolarization-activated variants (Zhao *et al*. 2004), *i.e.*, reversal of the polarity of the voltage-dependent activation. This observation suggests that the coupling between the VS domain and activation-gate can be modulated by subtle structural changes at their interface.

## Inactivation of the Na^+^ channels

Inactivation is one of the characteristic phenomena of all ion channels, and can be explained based on the above structural and thermodynamic discussions. While a transport rate of 10^7^ ions per second per channel might be achieved in electrophysiology experiments, such a high rate of continuing transport is unsustainable for any living cell. Under physiological conditions, the transport in depolarization-activated K^+^ channels is mainly driven by the Nernst potential of K^+^ ions but against ∆*Ψ*_M_. Thus, cells are protected from depletion by negative feedback of these channels. In contrast, because their ion flows reduce ∆*Ψ*_M_, depolarization-activated Na^+^ (and Ca^2+^) channels usually function in a positive feedback manner. Therefore, a Na^+^ channel must be equipped with an inactivation mechanism to quickly auto-deactivate itself after activation. More specifically, Na^+^ channels from both eukaryotic and prokaryotic cells undergo a slow inactivation, with a time constant in the range from tens to hundreds of millisecond (Zarrabi *et al*. 2010). In addition to this mechanism, eukaryotic Na^+^ channels often exhibit fast inactivation (with a time constant in the millisecond range) (Bagneris *et al*. 2015), which is so fast that an ensemble of the channels has not reached its “true” maximum current (*I*_max_) before all channels are closed (Schmidt *et al*. 2009). In addition, certain types of K^+^ channels (*e.g.*, the Shaker *K*_V_ channels) and hyperpolarization-activated channels (*e.g.*, HCNs) also exhibit inactivation in the presence of prolonged activation cues (Hoshi *et al*. 1991; Rothberg *et al*. 2002). Many mutational inherited diseases that target VGICs are shown to impair the inactivation function (Catterall 2010).

Several hypotheses on the mechanisms of inactivation for ion channels have been postulated, including collapse of the selectivity-filter, blocking of the activation-gate by tethered polypeptides, reshaping of the activation-gate, and allosteric blocking (Bagneris *et al*. 2015; Cuello *et al*. 2010b; Payandeh *et al*. 2012; Yan *et al*. 2017). While each model is supported by experimental data and may function in certain types of channels, the exact type of energetic driving forces for such inactivation and for later recovery from the inactive state remains to be clarified. In eukaryotic Na^+^ channels, fast inactivation after opening of the gate is achieved through releasing a tethered polypeptide (*i.e.*, the blocker) from an idle state and/or exposing a binding site for the blocker, thus leading to subsequent gate blockage. Such blockage is reversed immediately after the channel is reset back to its resting condition. For example, a cytoplasmic inactivation lid located between the third and fourth homologous 6TM repeats of the mammalian Na^+^ channel confers fast inactivation through a hinged-lid mechanism (Stuhmer *et al*. 1989). In addition, the Shaker *K*_V_ channel was shown to be plugged by its N-terminal polypeptide which acts as a blocker of the ion permeation pathway. If the N-terminus is truncated, inactivation is slowed down (Hoshi *et al*. 1990). This so-called N-type fast inactivation is thought to permit the channel to be available for quick re-activation, for example in the presence of high-frequency activation pulses (Hoshi *et al*. 1990). In comparison, slow inactivation (also called C-type inactivation) is affected by structural perturbations in the C-terminal region (including the S6 helix). However, N-type activation and C-type inactivation are not mutually exclusive, and have been shown to occur simultaneously in a number of channels (Hoshi *et al*. 1991).

Unlike fast inactivation, which is usually associated with channel-specific mechanisms, slow inactivation is more likely to be used as a common mechanism across different channels. Here, we will discuss Na^+^ channels lacking fast inactivation as an example to illustrate such a common mechanism. After prolonged activation under a persistent activation cue, the Na^+^ channel gradually loses all of its conductivity. Under the resting condition, the deactivated channel takes certain time to return to the state capable to become fully activated again. This relaxation process is called recovery. In addition, after pre-treating the channel with a mild activation cue, the channel shows a reduced “*I*_max_” in response to a full-strength activation cue; this phenomenon is called desensitization. Interestingly, raising the concentration of substrate ions increases the recovery rate from slow inactivation (Townsend and Horn 1997), suggesting that whatever blocks the gate during slow inactivation competes with the substrate ions. Single-channel recordings (*e.g.*, in Hoshi *et al*. 1991) showed that the slow inactivation is associated with a suddenly reduced opening frequency but not a gradually lowered current (or *g*_sc_) for a single channel.

**Fig. 4:**
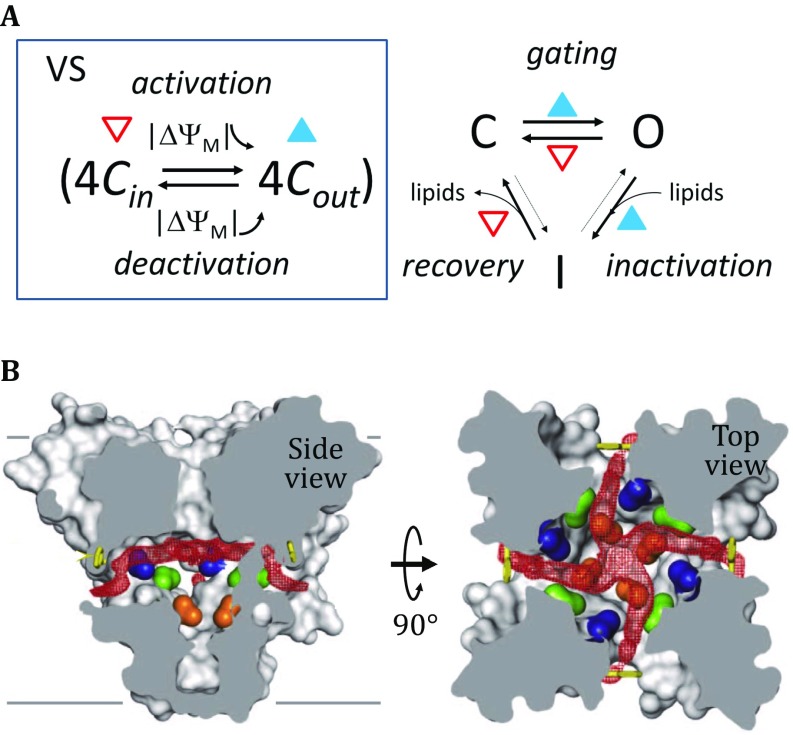
Putative mechanism of the slow inactivation of ion channels. **A** Kinetic model of the gating process. Represented in the box, the VS domain has a C_in_ state (open triangle) and a C_out_ state (filled triangle). On the right side, the activation-gate has a closed (C) state, open (O) state, and an inactivated (I) state. The state of the VS domain is coupled to that of the activation-gate. The thickness of the arrows indicates the rate of the corresponding reaction. **B** Fenestrations on the wall of the central pore in the closed state of Na_V_Ab/3RVY. Red electron densities correspond to lipid penetration paths. These paths are presumably enlarged upon the activation-gate is switched to the open state, thus allowing more rapid infiltration of lipid molecules into the gate pore. This panel is adapted from this figure in Payandeh *et al*. (2011)

It has long been proposed that blockers acting in channel inactivation may function through either hydrophobic or hydrophilic pathways (Hille 1977). Between these two routes, the hydrophobic path is more likely to be associated with slow inactivation as this path exhibits a slower diffusion rate. Here, we propose a model of reaction kinetics for the general lipid-infiltration-mediated mechanism in charge of the slow inactivation in Na^+^ channels (Fig. 4). In our model, the surrounding lipid molecules behave as reversible inhibitors (blockers) and penetrate into the activation-gate through interfaces between the subunits, thus deactivating the opened channel by blocking the activation-gate. This model is reminiscent of a model proposed before the first ion channel was cloned (Hille 1977). However, in comparison to the previous model, we provide a physical explanation for the process of slow inactivation. A comparable mechanism has also been used to explain the adaptation phenomenon in mechanosensitivity channels of small-conductance (MscS) (Zhang *et al*. 2016). Curiously, the slow inactivation observed in these two types of channels appears to follow similar pathways: both become inactivated only after the gate is opened; both are sensitive to mild activation cues; and both require considerable recovery time before restoring their ability to be activated again. In addition, the inactivation phenomenon of ion channels is qualitatively similar to reversible inhibition by certain toxic drugs (Schmidt *et al*. 2009). In fact, drug blocking and inactivation appear to enhance each other (Hille 1977). Both types of inhibition can be explained by structural changes at the activation-gate upon activation. In particular, the pore-lining S6 helices become loosely packed with each other once the gate is opened, allowing surrounding lipid molecules to infiltrate slowly into the gate pore. Such penetration will result in reduced pore size and increased hydrophobicity of the gate pore, thus decreasing the open probability of the gate by virtual of the dry/wet two-state mechanism in which a constant *g*_sc_ is assumed for the single channel. In contrast to the sponge-like behavior observed for the open pore in lipid absorption, the closed pore slowly squeezes out the plugging lipid molecules in the recovery process upon withdrawing the activation cue. In addition, the diffusion rate of lipid molecules into the activation-gate is likely to be positively correlated with the size of the gate. Thus, the rate of slow inactivation depends on activation cues. In particular, the lipids may infiltrate into the activation-gate even before it enters its wet state. Therefore, pre-activation with mild activation cues results in a partial filling of the gate pore (or a fraction of the channel ensemble) with lipid molecules, thus reducing the probability of gate opening. Together, these arguments should explain how desensitization is achieved by the lipid-infiltration mechanisms.

In agreement with our hypothesis, in the closed-state structure of Na_V_Ab/3RVY, four lateral portals (termed fenestrations) are found on the wall of the central cavity, connecting the lipid bilayer to the interior of the central pore (Payandeh *et al*. 2011). These fenestrations were unexpectedly penetrated by fatty acyl chains that extended into the central cavity (Fig. 4B). The portals are suspected to serve as the entries for lipophilic inhibitors, including local anesthetics. Importantly, these portals become enlarged in the open state of the activation-gate (McCusker *et al*. 2012), presumably allowing more rapid penetration of lipid molecules into the central pore. Similarly, in the crystal structure of Na_v_Rh/4DXW, a lipid/detergent molecule is observed in the central cavity, and in the crystal structure of Na_V_1.4/5XSY from electric eel (EeNa_V_1.4), the activation-gate was found to be “kept open” by a detergent-like molecule inside the gate (Yan *et al*. 2017). Moreover, changing the hydrophobicity and saturation properties of lipid molecules of the membrane bilayer also affects the rate of slow inactivation (Schmidt *et al*. 2009). Also in line with our hypothesis of lipid-infiltration-mediated inactivation, mutations in the middle of S6 near the fenestration (*e.g.*, I1575A in repeat-IV of rNa_V_1.4) were shown to increase the rates of both inactivation and recovery (Zarrabi *et al*. 2010), probably by virtual of reducing kinetic barrier for lipid infiltration. A single-point mutation at a conserved Asn residue in S6 alters slow inactivation in Na_V_ channels (Wang and Wang 1997). In contrast to our hypothesis, the model of collapsed selectivity-filter for slow inactivation would predict gradually reduced *g*_sc_ value in the single-channel recording, which strongly contradicts presently available experimental observations (Cuello *et al*. 2010a).

The pore-lining S6 helices of Na^+^ channels usually maintain a degree of helix bundle crossing that is lower than those found in inactivation-resisting K^+^ channels. In the open state, the C-terminal ends of S6 helices become more flexible than the closed state (*e.g.*, in Na_V_Ms/3ZJZ), allowing formation of dynamic portals between subunits. In addition, subunits of bacterial Na^+^ channels often contain C-terminal domains as an extension of S6, which are further tetramerized in the cytosol. In crystal structures of closed gates (*e.g.*, in Na_V_Ae/4LTO (Shaya *et al*. 2014)), this cytosolic tetrameric region, in particular the linkers between S6 helices and C-terminal helix bundle, is well ordered; however, it becomes (partially) disordered in the open state, presumably permitting formation of lateral portals on the wall of the activation-gate. The rate of inactivation in Na_V_Ms is also positively related to the presence of the flexible linker and, in particular, of three consecutive acidic residues in this region (Bagneris *et al*. 2013). Presumably, the repulsion between these concentrated (3 × 4) acidic residues maintains the lateral portals of the gate, thus permitting inactivation to proceed. Another channel whose crystal structure (3EFD) has been reported to show straight (rather than crossing) pore-lining helices is the full-length channel KcsA of *S. lividans*. If its cytosolic C-terminal four-helix bundle region is stabilized following Fab binding, the inactivation rate is reduced (Uysal *et al*. 2009). In contrast, in inactivation-resisting voltage-gated K^+^ channels, the C-terminal regions of S6 helices are more curved and tilted (*e.g.*, in the crystal structure of Kv1.2/2A79), forming a highly crossing, right-handed bundle. This more twisted helix bundle is likely to resist lipid penetration, in a manner similarly to the MscL-type of mechanosensitivity channels (Zhang *et al*. 2016). In addition, subunits of K^+^ channels often contain an N-terminal domain which is tetramerized in cytosol “underneath” the activation-gate (*e.g.*, in Kv1.2/2A79), presumably constricting the overall flexibility of the channel tetramer. These structural features may contribute to the lack of inactivation in certain K^+^ channels. Moreover, the acidic residue introduced by the above-mentioned K1237E mutation in the filter region of repeat-IV of rNa_V_1.4 (Todt *et al*. 1999) is subjected to an extra electrostatic force, compared with the wild-type channel. This electrical force shifts the central pore towards the extracellular direction. Together with the hydrophobic mismatch, this outward force is likely to make the pore-lining helices of the activation-gate more compact, thus reducing the penetration probability of surrounding lipid molecules and resulting in ultra-slow inactivation (Todt *et al*. 1999). Taken together, dynamic inter-subunit portals between S6 helices in Na_V_ channels are likely to serve as the paths for lipid molecules to penetrate into and exit from the central pore during inactivation and recovery, respectively.

## Concluding remarks

The microenvironment provided by the cellular membrane, including lipid bilayer and the carried electrostatic membrane potential, plays essential roles in ensuring proper functioning of ion channels. The thermodynamic interactions between channels and their membrane environments are keys to understanding of the molecular mechanisms of channel operation. However, by emphasizing the common mechanisms of VGICs, we do not suggest that individual details and mechanisms are of lesser importance to the overall knowledge on ion channels. Our message distilled here was formed through appropriate abstraction, and is based on the assumption that all functions of membrane proteins result from generally applicable physical principles. We anticipate that meaningful combination of quantitative thermodynamic consideration with detailed structural studies as well as biochemical and functional analyses will improve our understanding of the general principle of ion channel operation as well as the detailed mechanisms of individual molecular targets.

## Electronic supplementary material

Below is the link to the electronic supplementary material.

Supplementary material 1 (PDF 153 kb)

## Abbreviations

HCN: Hyperpolarization-activated cyclic nucleotide-gated channel
KcsA: K^+^ channel from *Streptomyces lividans*
K_V_AP: Voltage-gated K^+^ channel from *Aeropyrum pernix*
Na_V_Ab: Voltage-gated Na^+^ channel from *Arcobacter butzleri*
Na_V_Ae: Voltage-gated Na^+^ channel from *Alkalilimnicola ehrlichei*
Na_V_Ms: Voltage-gated Na^+^ channel from *Magnetococcus spirillium*
Na_V_Rh: Voltage-gated Na^+^ channel from *Rickettsiales* sp.
TM: Transmembrane (helix)
TPC: Two-pore channel
VGIC: Voltage-gated ion channel
VS: Voltage sensor
