# Supplementary material for: Thermodynamics of voltage-gated ion channels
Source: Biophys Rep. 2018 Nov 16;4(6):300–19. doi: 10.1007/s41048-018-0074-y (PMC6276078; doi:10.1007/s41048-018-0074-y)
Supplement: Supplementary file 1 — Supplementary material 1 (PDF 153 kb) [file 41048_2018_74_MOESM1_ESM.pdf]

Supplementary material

Gating-charges

In the two-state model of a voltage-sensor, gating-charge(s) ΔQ is the most important characteristic parameter. In a simple case, the VS domain contains one point-electric charge, q. The gating-charge is defined as

$$\Delta Q \equiv q(\alpha_1 - \alpha_0), \tag{S1}$$

where subscripts 0 and 1 represent the two states (*e.g.*, C_{in} and C_{out}); and α_i is termed the state-dependent sensing coefficient. In first approximation, α_i is the fraction ratio of the length between the point charge and the protein surface on the reference side (*e.g.*, the cytosolic side) of the membrane relative to the overall thickness of the focused electrical field of $\Delta\Psi_M$, at the ith state (see Fig. S2). For instance, if the charge remains on the same side of $\Delta\Psi_M$, it contributes zero to the gating-charge; if it moves across the entire electrical field of $\Delta\Psi_M$, the charge contributes q units to the overall gating-charge; and if it moves half way across the field of $\Delta\Psi_M$, the charge contributes ½ of the q electron units.

For a VS domain containing multiple charges, the gating-charge is defined as

$$\Delta Q \equiv \sum_j q_j\,(\alpha_{j1} - \alpha_{j0}), \tag{S2}$$

where the summation is over all of the positive and negative charges. For a more precise mathematical definition of ΔQ, see Islas and Sigworth (2001). Only those charges that show a large change in their sensing coefficients between the two states contribute significantly to the overall gating-charges. Paired electric charges are likely to (partially) cancel their contributions to the overall gating-charges.

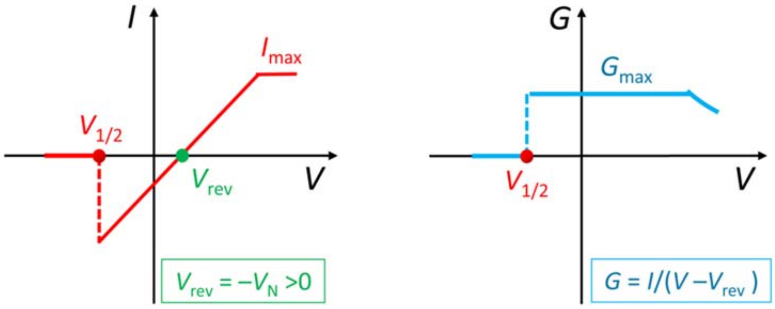

Fig. S1 I–V and G–V curves of an ideal ion channel. In an ideal depolarization-activated ion channel, it is assumed that activation occurs in a very narrow range of voltage (V) around $V_{1/2}$ (*i.e.* $\Delta Q >> 0$) and that the (V_{rev}-adjusted chord) conductance, G, of the open channel is constant before the current reaching saturation (I_{max}). In addition, $V_{1/2} < 0$ (*i.e.*, $\Delta G^0 < 0$), $V_{\mathrm{rev}} >0$ (*i.e.*, $V_{\mathrm{N}} < 0$), and inactivation phenomenon is not included. In the electrophysiology field, $-V_{\mathrm{N}}$ is often referred to as reversal potential V_{rev}. For a real channel, both the I–V and G–V curves become smoothened. Note that the chord conductance is different from the "slope" conductance, G_{slope} ($\equiv \mathrm{d}I/\mathrm{d}V$), for a non-linear I–V curve

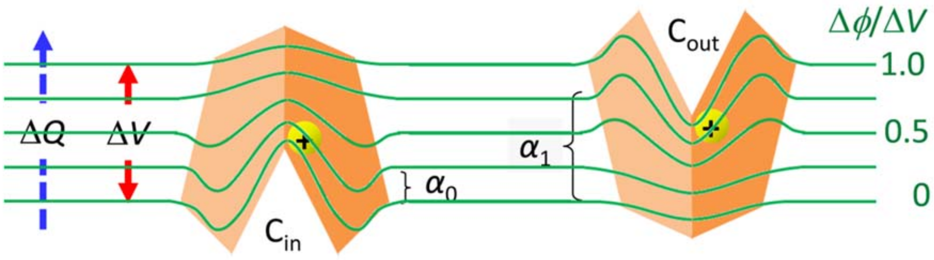

Fig. S2 Schematic diagram of a single gating-charge. A voltage sensor (VS) possesses distinct surface-exposed crevices in its C_{in} and C_{out} conformation. The "external" voltage (V) is applied by the cell (or experimentally) to the membrane bilayer as well as the embedded VS. Because of the crevice, the electrostatic potential (ϕ) is not uniformly distributed inside the protein. According to Eq. 3, changing of the external voltage (ΔV) reshapes the ϕ distribution. It is this $\Delta\phi$ (represented by the *green lines*—contour surfaces in 3D) that is sensed by electrically charged group(s) (represented by the *yellow sphere*). In this diagram, we arbitrarily choose the cytosolic surface of the membrane as the reference point for both ΔV and $\Delta\phi$
